# Plasma level of human epididymis protein 4 is associated with risk of future venous thromboembolism—the Trøndelag Health study

**DOI:** 10.1016/j.jtha.2026.03.009

**Published:** 2026-03-18

**Authors:** Celina Janene Cathro, Ellen-Sofie Wulff Eilertsen, Therese H. Nøst, Kristian D. Hindberg, Weihong Tang, Weihua Guan, Aaron R. Folsom, Christian Jonasson, Laurent Thomas, Kristian Hveem, Sigrid K. Brækkan, John-Bjarne Hansen

**Affiliations:** 1 Thrombosis Research Group, Department of Clinical Medicine, UiT – The Arctic University of Norway, Tromsø, Norway; 2 Division of Internal Medicine, Thrombosis Research Center, University Hospital of North Norway, Tromsø, Norway; 3 Department of Public Health and Nursing, HUNT Center for Molecular and Clinical Epidemiology, Norwegian University of Science and Technology, Trondheim, Norway; 4 HUNT Research Center, Norwegian University of Science and Technology, Levanger, Norway; 5 Levanger Hospital, Nord-Trøndelag Hospital Trust, Levanger, Norway; 6 Department of Community Medicine, UiT – The Arctic University of Norway, Tromsø, Norway; 7 Division of Epidemiology and Community Health, University of Minnesota, School of Public Health, Minneapolis, MN, USA; 8 Division of Biostatistics, University of Minnesota, School of Public Health, Minneapolis, MN, USA; 9 Department of Clinical and Molecular Medicine, Norwegian University of Science and Technology, Trondheim, Norway; 10 BioCore – Bioinformatics Core Facility, Norwegian University of Science and Technology, Trondheim, Norway; 11 Clinic of Laboratory Medicine, St. Olavs Hospital, Trondheim, Norway; 12 Department of Research, St. Olavs Hospital, Trondheim, Norway

**Keywords:** deep vein thrombosis, human epididymis protein 4, plasma, pulmonary embolism, venous thromboembolism

## Abstract

**Background:**

Plasma human epididymis protein 4 (HE4) levels have been associated with the risk of future venous thromboembolism (VTE) in a proteome-wide study.

**Objectives:**

We aimed to investigate whether plasma HE4 levels were (i) correlated with growth differentiation factor-15 (GDF-15), a marker of oxidative stress, (ii) associated with VTE risk after adjustment for potential confounders, and (iii) genetically regulated, as assessed by protein quantitative trait loci analyses.

**Methods:**

A case-cohort was derived from the Trøndelag Health Study (*N* = 50 800), including 294 incident VTE cases over 5 years of follow-up and a randomly sampled age- and sex-weighted subcohort (*n* = 1066). HE4 and GDF-15 levels were measured using an aptamer-based method in plasma collected at baseline. Weighted Cox regression was used to estimate hazard ratios for VTE, with 95% CIs based on quartiles (Q1–4) of HE4 levels.

**Results:**

HE4 levels correlated with GDF-15 levels (r = .71; *P* = 3.42 × 10^−211^), and both proteins were elevated in smokers. Participants with HE4 levels in the highest quartile (Q4) had a 2.5-fold higher risk of future VTE (hazard ratio, 2.54; 95% CI, 1.60–4.03) than those in the lowest quartile (Q1), after adjustment for age, sex, and sample batch. The risk remained after multivariable adjustments. Two genetic variants in *trans*, with genome-wide significance, explained only 1.6% of the variability in plasma HE4.

**Conclusion:**

HE4 levels were associated with an increased risk of future VTE after adjustment for major confounders and did not appear to be strongly genetically regulated. Our findings suggest that HE4 levels may reflect an underlying condition, eg, oxidative stress, that predisposes to VTE.

## 1 | INTRODUCTION

Venous thromboembolism (VTE), including deep vein thrombosis (DVT) and pulmonary embolism (PE) [1], affects 1 in 12 people over their lifetime [2,3] and almost 10 million people worldwide annually [1]. Physical complications from VTE include postthrombotic syndrome, post-PE syndrome, recurrence, and death [4–6]. In addition, VTE patients are at a more than 2-fold higher risk of developing depression [7] and receiving disability pension [8] compared with the general population. The observed rise in VTE incidence over the last few decades [9,10] is expected to continue due to an increase in several major VTE risk factors, including cancer [11,12], obesity [13,14], and aging populations [15,16]. Therefore, it is imperative to discover novel biomarkers to improve risk stratification and pursue targeted VTE prevention.

Previous studies indicated that human epididymis protein 4 (HE4) levels, also known as WAP 4-disulfide core domain protein 2 (WFDC2) [17], could be a risk factor for VTE risk in selected patient groups with a high VTE risk, such as patients with epithelial ovarian cancers [18] and women who underwent gynecologic laparoscopic surgery [19]. Recently, we reported that plasma HE4 levels showed a Bonferroni-significant association with the risk of future VTE in an unsupervised proteome-wide discovery study using a case-cohort sample derived from the general population [20].

HE4 is upregulated and secreted under oxidative stress [21,22] and is upregulated in various cancers [23–25]. In addition, HE4 is elevated in some inflammatory diseases [22,26,27] and is associated with cardiovascular disease (CVD) history in chronic obstructive pulmonary disease (COPD) patients [28]. Various factors, such as age, sex, body mass index (BMI), smoking status, and kidney function, are associated with serum HE4 levels [21,29,30]. Moreover, HE4 levels are strongly correlated with growth differentiation factor-15 (GDF-15) levels, a marker of oxidative stress [31,32], in patients with heart failure [33]. High plasma GDF-15 levels are associated with an elevated risk of future VTE [34,35], whereas genetically predicted GDF-15 levels are not associated with risk of VTE [34], implying that the observed relationship between GDF-15 and VTE is unlikely to be causal.

As the interrelation between plasma HE4 and GDF-15 levels and the association between HE4 levels and VTE risk have been scarcely explored in the general population, we first aimed to explore whether HE4 levels correlate with GDF-15 levels and are influenced by smoking, a trigger of oxidative stress, in this population. Second, we aimed to investigate whether the association between plasma HE4 levels and VTE risk is confounded by shared factors, including age [15], sex [36], BMI [37], inflammation [38,39], CVD history [40,41], and impaired kidney function [42,43]. Third, we aimed to explore whether genetic variants explain variability in plasma HE4 levels by applying protein quantitative trait loci (pQTL) analysis to further assess the relationship between HE4 and VTE risk from a causal perspective.

## 2 | METHODS

### 2.1 | Study population – the third Trøndelag Health Study survey (HUNT3)

The study population was derived from the third survey of the Trøndelag Health Study (HUNT3), which is a population-based cohort conducted in the (former) Nord-Trøndelag County from 2006 to 2008 [44,45]. All residents aged ≥20 years were invited, and 50 800 individuals participated (response rate of 54.1%). Participants were followed for 5 years from the date of inclusion in the cohort, and all incident VTE cases were recorded. The study was approved by the Regional Committee for Medical and Health Research Ethics, and all participants provided written informed consent to participate and to the use of their data for medical research.

### 2.2 | Baseline information, measurements, and blood sampling in HUNT3

Participant information was collected at cohort inclusion by physical examination, self-administered questionnaires, and blood samples. Nonfasting blood samples from an antecubital vein were collected into vacutainer tubes containing EDTA as an anticoagulant. Following centrifugation (2500 × *g* for 10 minutes at 6 °C), plasma was stored at −80 °C in the Trøndelag Health Study (HUNT) biobank in Levanger, Norway. Information on age, sex, daily smoking (yes/no), and history of arterial CVD (angina pectoris, stroke, and myocardial infarction) was obtained from self-administered questionnaires. Weight (to the nearest 0.5 kg) and height (to the nearest centimeter) were measured with participants wearing light clothing and no shoes, and BMI (kg/m^2^) was calculated as weight in kilograms (kg) divided by height in meters squared (m^2^). C-reactive protein (CRP) levels were measured using a latex immunoassay method (Abbott Clinical Chemistry) [46]. Serum creatinine was measured using the alkaline picrate method (Abbott Clinical Chemistry), recalibrated from the Jaffe to the enzymatic method, and the estimated glomerular filtration rate (eGFR) was assessed using the Chronic Kidney Disease Epidemiology Collaboration equations [47].

### 2.3 | Outcome registration and validation of VTE

Incident VTE cases were identified by searching hospital discharge diagnosis and autopsy registries for relevant International Classification of Diseases, 10th Revision codes (126.0, 126.9, 167.6, 180.0–180.3, 180.8, 180.9, 181, 182.0–182.9, O22.3, O22.5, O87.1, and O87.3), covering the three hospitals within the study catchment area (Namsos Hospital, Levanger Hospital, and St. Olav’s Hospital [Trondheim]) from inclusion through the end of 2019. The medical records of each participant with a potential VTE event were reviewed by trained health care professionals, and VTE was confirmed when clinical signs and symptoms of PE and/or DVT were documented, radiological procedures (ultrasound, ventilation/perfusion scan, venography, or computed tomography pulmonary angiogram) or autopsy confirmed the diagnosis, and treatment was initiated (unless there were contraindications or immediate death). If a participant had both DVT and PE, the event was categorized as PE. A standardized form was used to note any additional information from the medical records of VTE cases regarding potential provoking factors and clinical risk factors up to 3 months prior to the VTE event.

### 2.4 | Study design

**FIGURE 1:**
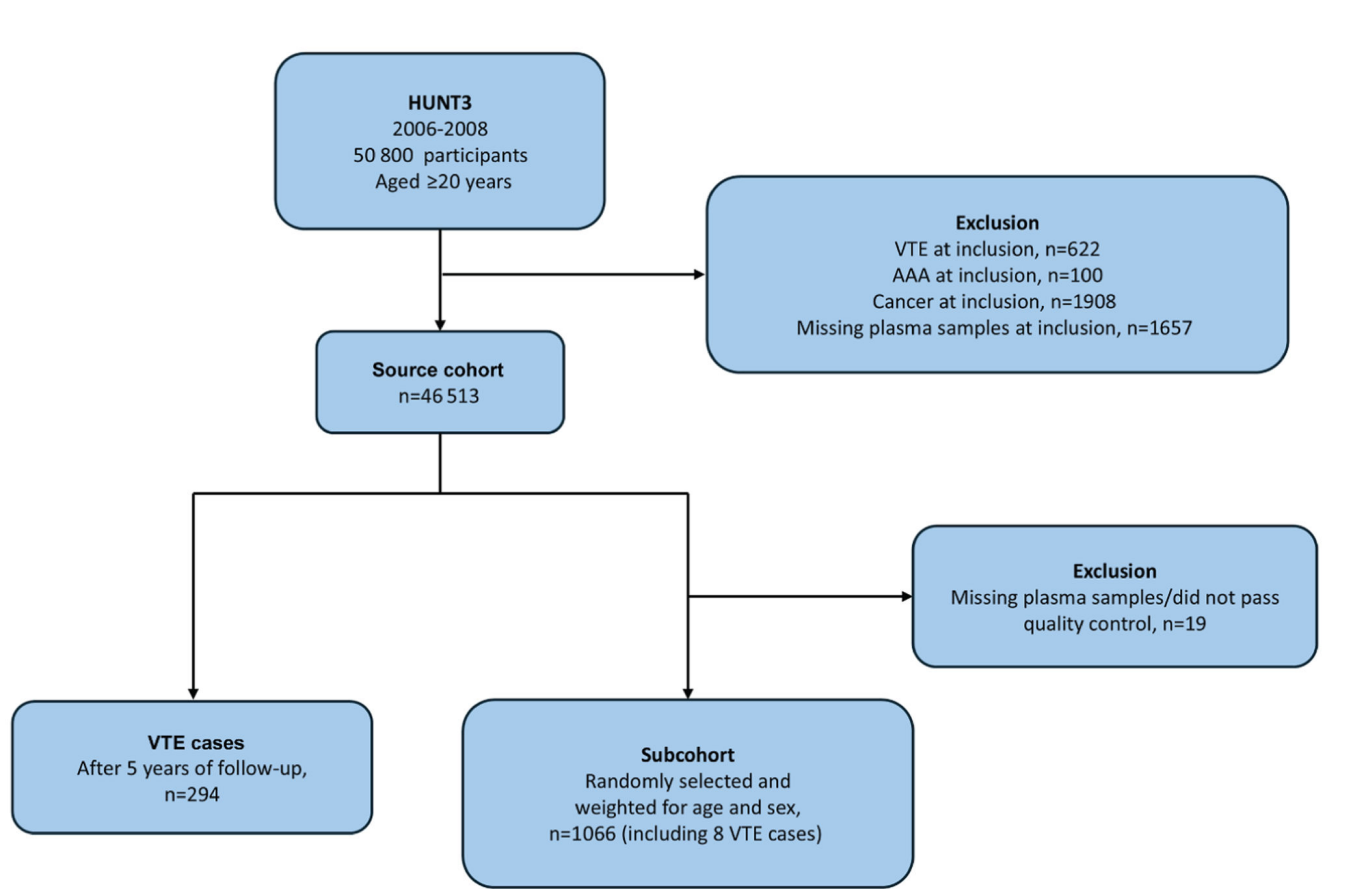
Flowchart of the study population. The flowchart illustrates the case-cohort design that was derived from the third survey of the Trøndelag Health Study (HUNT3; 2006–2008). AAA, abdominal aortic aneurysm; VTE, venous thromboembolism.

This case-cohort study included all incident VTE cases occurring within the first 5 years of follow-up and a randomly sampled subcohort from the HUNT3 survey (*N* = 50 800), as illustrated in the study population flowchart (Figure 1) [20]. Participants with prevalent abdominal aortic aneurysm (*n* = 100), VTE (*n* = 622), cancer (*n* = 1908), and those with missing plasma samples (*n* = 1657) were excluded from the HUNT3 source population prior to baseline inclusion. Among the 46 513 participants eligible for this study, 294 had an incident VTE during the first 5 years of follow-up and were included as cases. The subcohort (*n* = 1085) was randomly sampled from the source cohort based on the age and sex distributions of VTE cases. Sampling of the subcohort from the source cohort was completed prior to the selection of VTE cases that occurred within the first 5 years of follow-up (*n* = 294), and was based on all VTE cases (*n* = 910) that occurred during the entire follow-up period (from inclusion to December 31, 2019). Nineteen samples from the subcohort were missing or did not pass quality control for protein measurement and were excluded, leaving 294 incident VTE cases and 1066 subcohort members in the final analytical sample, of whom 8 were cases because of the sampling method.

### 2.5 | Measurements of plasma HE4 and GDF-15

In 2022, plasma samples from VTE cases and the subcohort were retrieved from the HUNT Biobank and shipped to SomaLogic Inc for analysis [48]. The SomaScan 7k aptamer-based platform (SomaLogic Inc) was used for semiquantitative measurements of HE4 and GDF-15 according to the manufacturer’s standard protocol [49]. The total coefficients of variation (CVs) for HE4 and GDF-15 in human plasma, as reported by SomaLogic, were 5.3% and 8.3%, respectively [50].

### 2.6 | Genotyping

Genotyping of the HUNT3 cohort was carried out using 1 of 3 Illumina HumanCoreExome arrays: HumanCoreExome12 v1.0, HumanCoreExome12 v1.1, or UM HUNT Biobank v1.0 [51]. Genotype calling was performed using GenTrain v2.0 in GenomeStudio v2011.1 (Illumina). Samples with a <99% genotype call rate, large chromosomal copy number variants, contamination >2.5% (estimated using BAFRegress [52]), discordance between genotypic and phenotypic sex, or non-European ancestry were excluded. Genetic variants with a Hardy–Weinberg equilibrium *P* < .0001 were excluded. Imputation was performed on samples with recent European ancestry by phasing single-nucleotide polymorphisms (SNPs) with EAGLE v2.3 [53], and imputing genotypes using Minimac3 v2.0.1 [54] from a merged reference panel constructed from the Haplotype Reference Consortium panel (release v1.1) [55] and a local reference panel based on 2202 whole-genome sequences from HUNT participants, resulting in 24.9 million SNPs (R^2^ > .3).

### 2.7 | Statistical analyses

All data processing and statistical analyses were performed with R v4.3.1 (R Foundation for Statistical Computing). For all analyses, individual protein measurements of HE4 and GDF-15 from the SomaScan data were log_2_-fold-transformed and normalized to a mean of 0 and an SD of 1. The normalized values of HE4 and GDF-15 were visualized in a scatter plot, and the correlation between the 2 variables was determined using the Pearson method. The normalized levels of HE4 and GDF-15 according to smoking status (daily smoking, yes/no) were displayed as box plots, and differences in normalized HE4 and GDF-15 levels between smokers and nonsmokers were tested using a 2-sample *t*-test.

The study population was divided into quartiles (Q1–4) based on cutoff values observed for plasma HE4 levels in the subcohort. The baseline characteristics of the subcohort across quartiles of HE4 levels were presented using descriptive statistics and expressed as percentages for categorical variables and as mean ± SD or median (IQR) for continuous variables.

For each participant, person-time of follow-up was calculated from the date of inclusion until the first VTE, migration, death, or the end of follow-up (5 years from the inclusion date), whichever occurred first. Weighted Cox proportional hazards regression models were used to estimate hazard ratios (HRs) with 95% CIs for VTE across increasing quartiles (Q1–4) of HE4 levels, with the lowest quartile (Q1) serving as the reference category. We applied the R package “cchs” (weighted Cox model for case-cohort data with stratified subcohort selection) [56]. We performed analyses of overall VTE and VTE subtypes, ie, unprovoked VTE, provoked VTE, DVT, and PE. Each analysis included 3 models. Model 1 was adjusted for age, sex, and sample batch; model 2 was adjusted for model 1 plus BMI; and model 3 was adjusted for model 2 plus eGFR, CVD history, CRP, and smoking. The proportion of missingness was low (<1%), and complete-case analyses were performed (the total number of individuals ranged from 1352 in model 1 to 1341 in model 3). Since eGFR may be measured with some error and HE4 is influenced by renal impairment, we also checked for an interaction between eGFR and HE4. In order to assess how strong an unmeasured confounder would need to be to explain the observed HR, we calculated the E-value [57] based on model 3, with overall VTE as the outcome and Q4 of HE4 as the exposure.

A generalized additive regression plot was created to assess possible nonlinearity between plasma HE4 levels and the risk of future incident VTE, adjusting for age, sex, sample batch, BMI, eGFR, CVD history, and CRP. Since HE4 is associated with cancer, plasma HE4 levels could potentially be influenced by occult cancer at baseline. We therefore performed sensitivity analyses in which we postponed the start of follow-up by 1 or 2 years; consequently, all VTEs occurring in the first 1 or 2 years would be excluded from these analyses.

The genome-wide dataset contained 24.9 million variants after imputation (R^2^ > .3) and 11.8 million variants after minimum allele frequency filtering (>0.0026). pQTL analysis was performed to identify genetic variants associated with the regulation of plasma HE4 levels using data from 1900 individuals with measured plasma HE4 levels obtained with the SomaScan platform (ie, those with available genome wide association studies data among the 910 VTE cases and 1066 subcohort members from the full follow-up). This pQTL analysis was performed in a genome-wide setting using BOLTLMM v2.4 [58] on rank-based inverse-normal-transformed outcomes, with age, BMI, VTE status, genotype batch, and the first 10 genetic principal components as covariates. The commonly used significance threshold of 5 × 10^−8^ was used to adjust for multiple testing. Genetic variants lying within ±500 kb of the *WFDC2* gene encoding HE4 were classified as “cis,” while variants outside this range were classified as “trans.” The *P* value results from the pQTL analysis were presented in a Manhattan plot, and the characteristics of the identified genome-wide significant SNPs were presented in a table. The variance of HE4 explained by the genome-wide significant SNPs was calculated using the following formula: 2 * AF * (1 − AF) * BETA^2^ / Var(Y), AF: allele frequency, BETA: beta coefficient/effect size and Var(y): total variance of the phenotype Y.

### 3 | RESULTS

**FIGURE 2:**
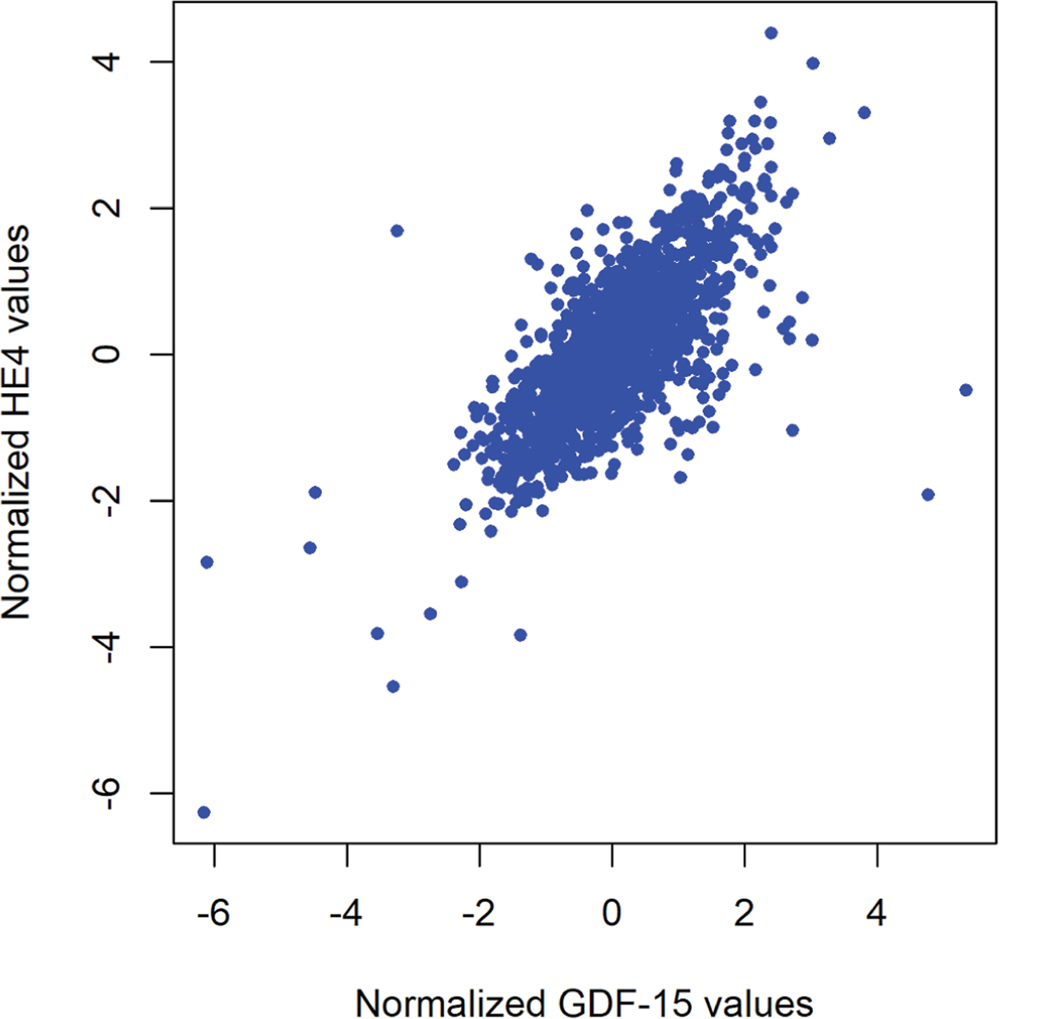
Scatterplot of normalized plasma human epididymis protein 4 (HE4) and growth differentiation factor-15 (GDF-15) levels. GDF-15 levels were strongly correlated with HE4 levels (r = .71; *P* = 3.42e-211). Plasma levels of HE4 and GDF-15 were log_2_-fold-transformed and normalized to a mean value of 0 and an SD of 1.

**FIGURE 3:**
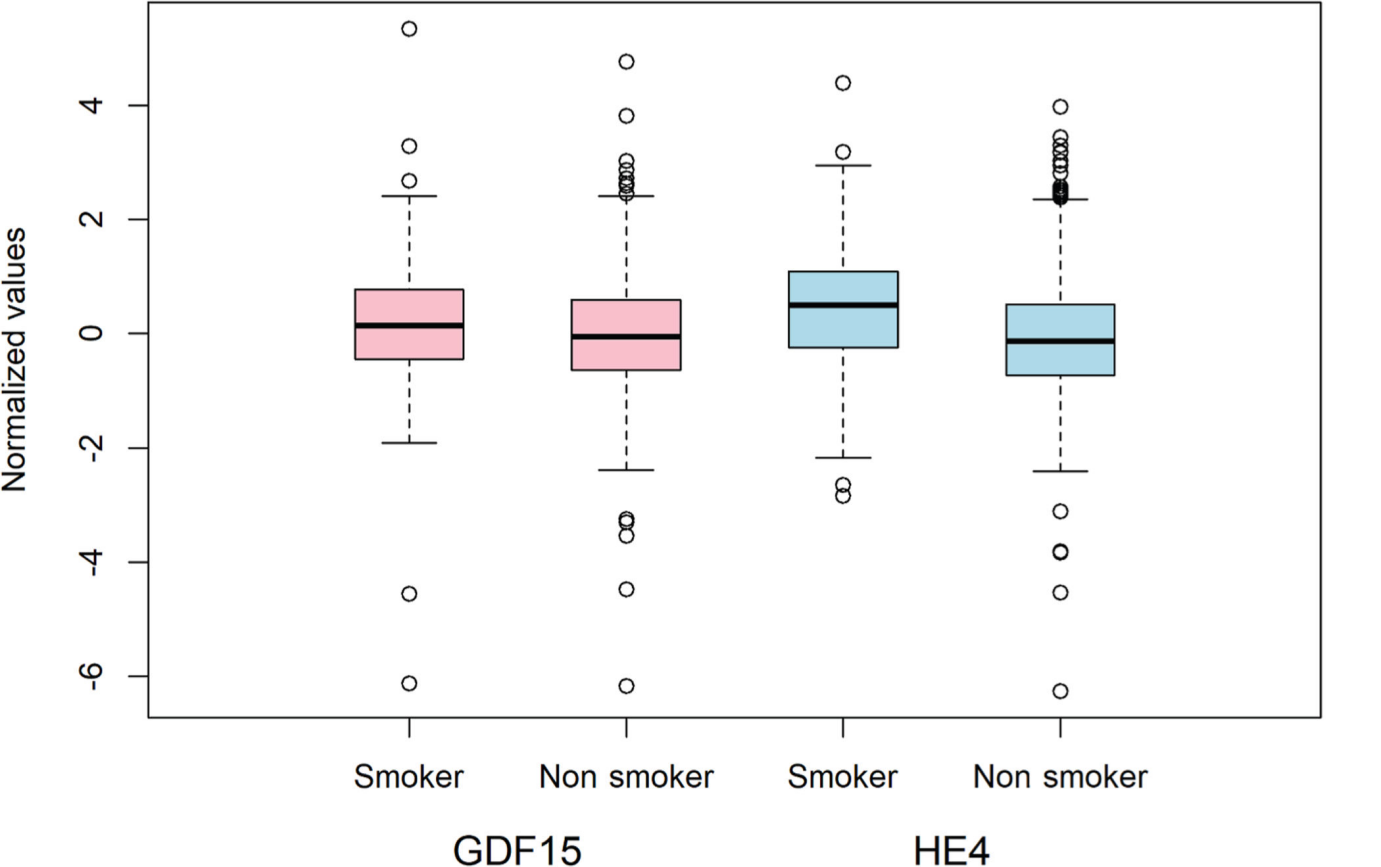
Box plots of normalized plasma human epididymis protein 4 (HE4) and growth differentiation factor-15 (GDF-15) levels according to smoking status (daily smoking, yes/no). Plasma HE4 and GDF-15 levels were significantly different between current smokers and nonsmokers (*P* = 8.56e-13 and *P* = 7.28e-3, respectively). Plasma levels of HE4 and GDF-15 were log_2_-fold-transformed and normalized to a mean value of 0 and an SD of 1.

The baseline characteristics of the subcohort members across quartiles of plasma HE4 levels are shown in Table 1. The mean age and median CRP levels increased, while the mean BMI and kidney function, as assessed by eGFR, decreased across higher quartiles of HE4 levels (Table 1). The proportion of smokers and those with a history of CVD increased across higher quartiles of HE4 levels. A strong correlation (r = .71; *P* = 3.42 × 10^−211^) was found between plasma HE4 and GDF-15 levels (Figure 2), and both were higher in daily smokers than in nonsmokers (*P* = 8.56 × 10^−13^ and *P* = 7.28 × 10^−3^, respectively; Figure 3). Baseline characteristics of VTE cases and subcohort members are shown in Supplementary Table S1.

Overall, the mean ± SD age was 66 ± 15 years in VTE cases and 64 ± 13 years in the subcohort (Supplementary Table S1). Furthermore, VTE cases had a higher BMI, slightly lower kidney function, and higher CRP, and consisted of more women than the subcohort members at inclusion (Supplementary Table S1).

The characteristics of participants with VTE (*n* = 294) at the time of diagnosis are shown in Supplementary Table S2. There were 113 incident DVTs (38%) and 181 PEs (62%). The majority of VTE events had 1 or more provoking factors (61%), and 19% of all VTE events were cancer-associated.

**FIGURE 4:**
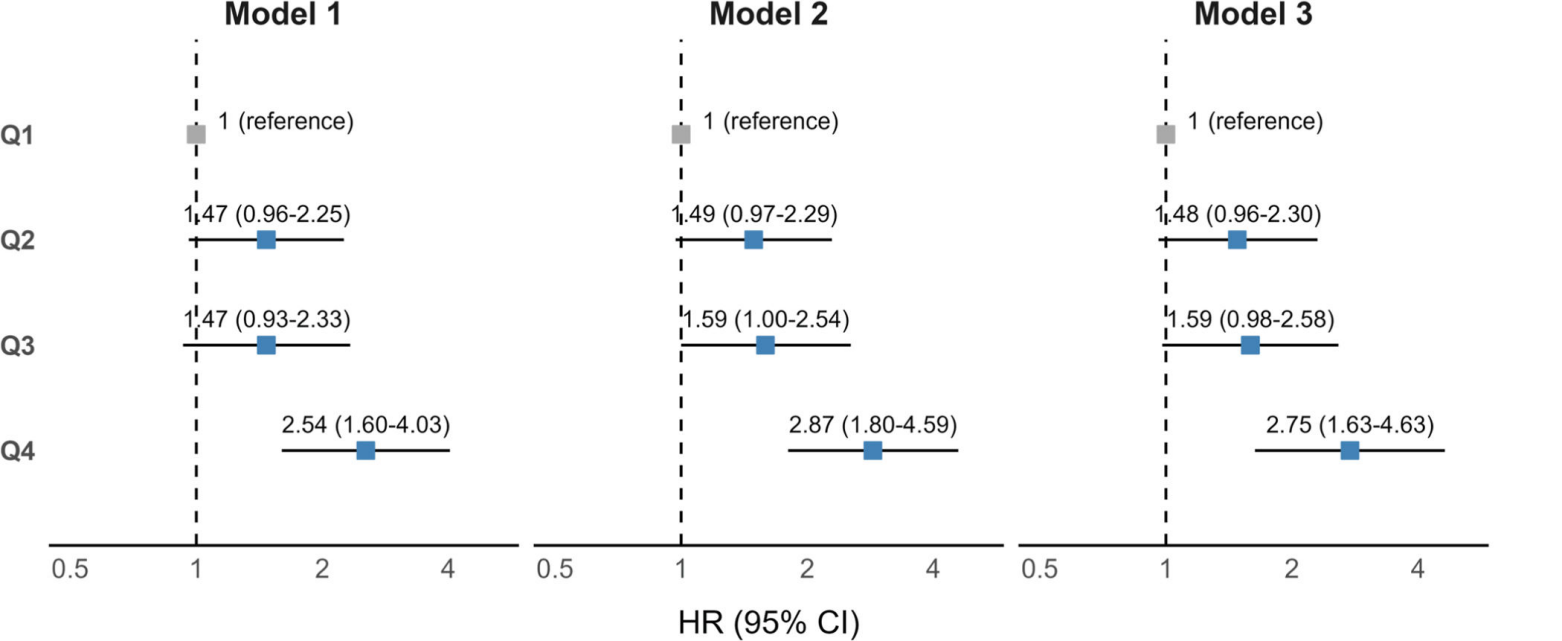
Forest plot displaying the hazard ratios (HRs) with 95% CIs for the risk of venous thromboembolism across quartiles (Q) of plasma human epididymis protein 4 levels, with the lowest quartile (Q1) used as reference. Model 1: adjusted for age, sex, and sample batch. Model 2: adjusted for model 1 plus body mass index. Model 3: adjusted for model 2 plus arterial cardiovascular disease history, estimated glomerular filtration rate, C-reactive protein, and smoking.

The HRs for future incident VTEs across quartiles of plasma HE4 levels are shown in Table 2 and Figure 4. There was a higher risk of overall VTE among individuals with plasma HE4 levels in the highest quartile (Q4) compared with those in the lowest (reference) quartile (Q1). In the model adjusted for age, sex, and sample batch (model 1), participants with plasma HE4 levels in Q4 had an HR of 2.54 (95% CI, 1.60–4.03) for overall VTE compared with those in Q1. Further adjustment for BMI (model 2) elevated the risk estimate for overall VTE (HR, 2.87; 95% CI, 1.80–4.59) in Q4 compared with Q1. After additional adjustment for eGFR, CVD history, CRP, and smoking status (model 3), the risk estimate for Q4 compared with Q1 remained higher than in model 1 (HR, 2.75; 95% CI, 1.63–4.63). The risk estimates for unprovoked and provoked VTE in model 1 were 3.11 (95% CI, 1.50–6.45) and 2.25 (95% CI, 1.30–3.90), respectively, for Q4 compared with Q1. After multivariable adjustment (model 3), the corresponding HRs were 3.29 (95% CI, 1.44–7.52) for unprovoked VTE and 2.43 (95% CI, 1.31–4.51) for provoked VTE.

**FIGURE 5:**
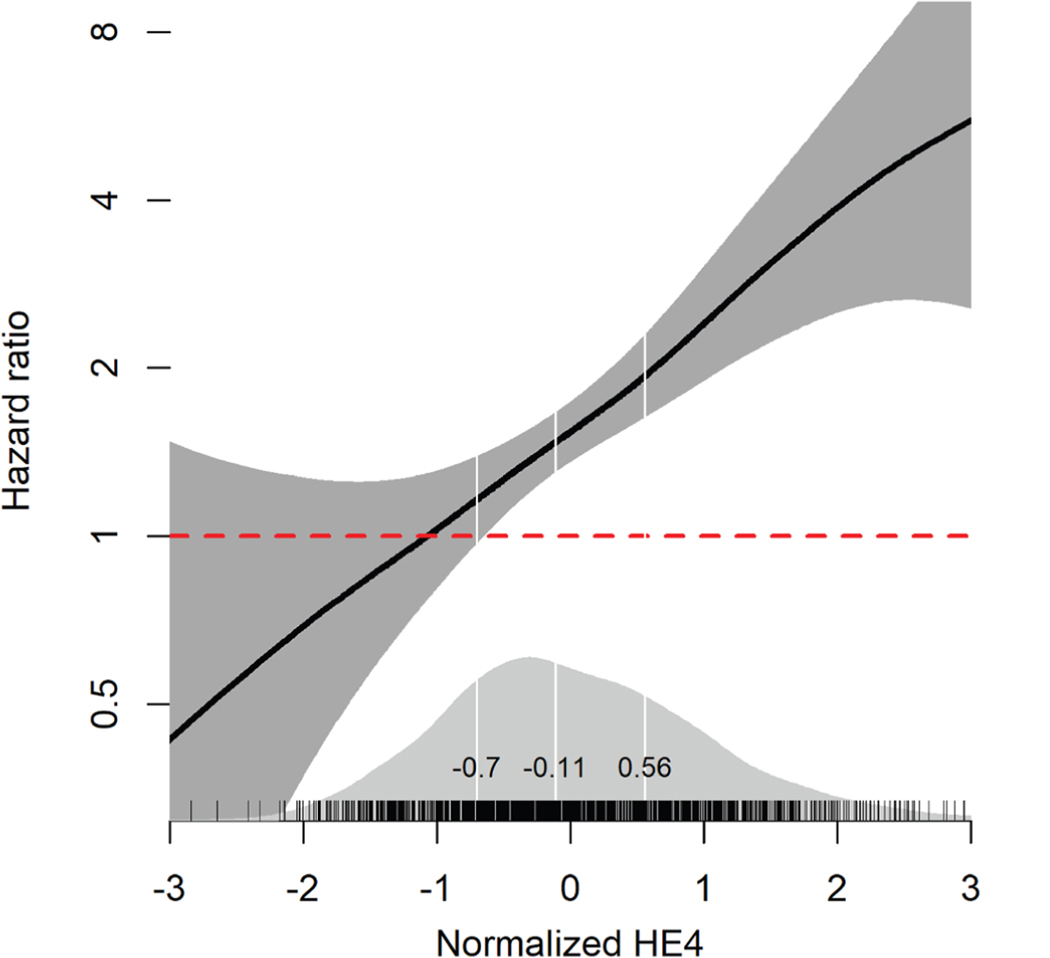
Spline plot displaying the hazard ratio (HR) of overall venous thromboembolism as a function of plasma human epididymis protein 4 (HE4) levels in a generalized additive regression model. The HRs were adjusted for age, sex, sample batch, body mass index, estimated glomerular filtration rate, cardiovascular disease history, and C-reactive protein. The black lines depict the HRs, while the white lines represent the quartile cutoffs. The dark gray areas represent the 95% CIs, while the light gray areas are density plots showing the distribution of plasma HE4 levels.

The association between plasma HE4 levels, assessed as a continuous variable, and the risk of overall VTE is shown in Figure 5. This figure shows a dose-response relationship between continuous plasma HE4 levels and the risk of future incident VTE. However, it is worth noting that the more extreme values in Q1 and Q4 may be less precise due to fewer events, resulting in wider 95% CIs. Sensitivity analyses with the start of follow-up 1 or 2 years after blood sampling did not change the results (Supplementary Figure S1).

The HRs for the DVT and PE subtypes are presented in Table 3. The HRs for DVT (3.51; 95% CI, 1.77–6.97) were higher than for PE (2.06; 95% CI, 1.17–3.62) when comparing the highest vs the lowest HE4 quartile in analyses adjusted for sample batch, age, and sex (model 1). Adjustment for BMI increased the risk estimates for both DVT and PE (model 2). After further adjustments for eGFR, CVD history, CRP, and smoking (model 3), the HRs were 3.04 (95% CI, 1.42–6.48) for DVT and 2.54 (95% CI, 1.32–4.85) for PE (Table 3). We found no interaction between eGFR and HE4 in any of the analyses. The CI was 2.36 for the E-value, indicating that an unmeasured confounder associated with both the exposure and the outcome, with risk ratios of at least 2.36 for each (conditional on the measured covariates included in model 3), would be required to move the 95% CI to include the null (HR = 1).

**FIGURE 6:**
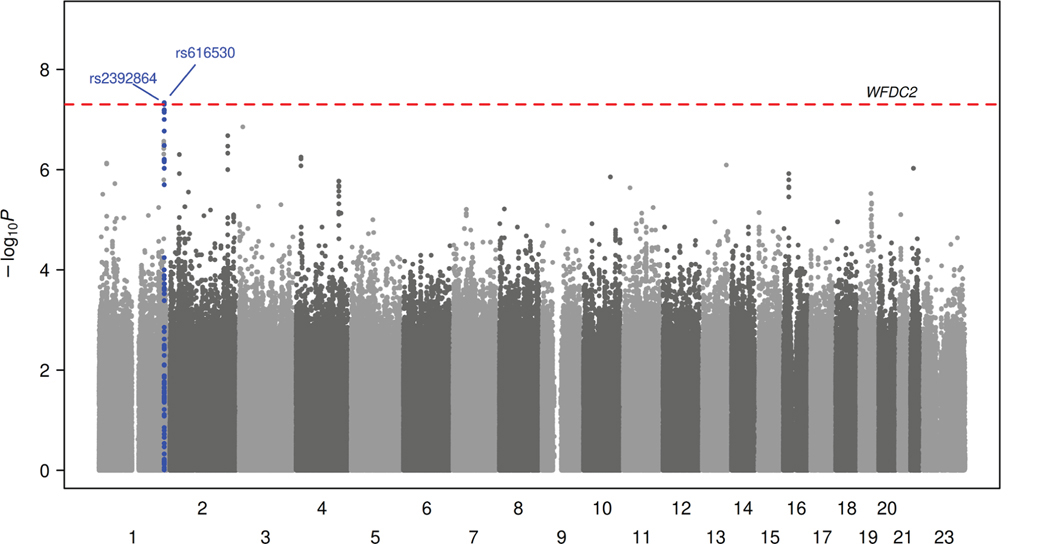
Manhattan plot of the protein quantitative trait loci analysis results (GRCh37/hg/19 was used as the reference human genome). The upper dashed red line indicates the genome-wide significance level threshold of *P* < 5 × 10^−8^ for associations with plasma variability in human epididymis protein 4 levels. Loci that reached genome-wide significance and nearby loci on chromosome 1 are highlighted in blue. The *WFDC2* gene, which encodes the human epididymis protein 4, is located on chromosome 20.

The results of the pQTL analysis are shown in Figure 6 and Supplementary Table S3. The pQTL analysis revealed 2 *trans-*pQTLs (rs616530 1:238727663_T/A and rs2392864 1:238726592_C/T) located in the intron region of LncRNA ENSG00000234464 on chromosome 1, with genome-wide significance for the regulation of plasma variability in HE4 levels (*P* = 4.6e-08 and *P* = 4.7e-08, respectively). These 2 SNPs were in high linkage disequilibrium (R^2^ = .968) and explained 1.6% (2 * AF * [1 − AF] * BETA^2^ / Var[Y] = 2 * 0.784456 * [1 − 0.784456] * 0.21753^2^ / 0.9998385 = 0.01600453) of the plasma variability in HE4. When conditioning on the lead SNP (rs616530), no genome-wide significant SNP was found (Supplementary Figure S2).

## 4 | DISCUSSION

In the present case-cohort study derived from the general population, plasma HE4 and GDF-15 levels were strongly correlated and varied in parallel across smoking status categories. We found a dose-response relationship between plasma HE4 levels and the risk of future incident VTE. Elevated HE4 levels displayed numerically higher risk estimates for unprovoked VTE and DVT than for provoked events and PE. In agreement with previous studies [21,28–30,59,60], we confirmed that elevated HE4 levels were associated with advancing age, arterial CVD history, current smoking, chronic inflammation (assessed by CRP), and impaired kidney function (assessed by eGFR). Importantly, the risk estimates for VTE by HE4 were not attenuated in the multivariable-adjusted model compared with the model adjusted for age, sex, and sample batch (model 1). We identified 2 *trans-*pQTLs with genome-wide significance that explained 1.6% of the variability in plasma HE4. Our findings suggest that plasma HE4 levels are a risk factor for future VTE and may partly reflect an underlying prothrombotic condition, such as oxidative stress.

HE4 has emerged as a promising new biomarker for various diseases, including the diagnosis and prognosis of ovarian cancer [61–63], improving the diagnostic performance of risk assessment models for ovarian cancer [64–68]. In addition, HE4 levels have been identified as a novel prognostic biomarker of heart failure severity [33,69] and recurrence of atrial fibrillation after catheter ablation [70]. HE4 levels are elevated in patients with COPD [22], cardiovascular complications in COPD [28], and chronic kidney disease [30].

A few studies have investigated the association between HE4 levels and VTE in selected patients at high risk of incident VTE [18,19]. In a cohort of 208 patients with epithelial ovarian cancer, 31 developed VTE during a median of approximately 2 years of follow-up [18]. Epithelial ovarian cancer patients who developed VTE had higher serum HE4 levels (median, 793.3 pmol/L; IQR, 196.6–835.6) compared with the overall cohort (median, 627.8 pmol/L; IQR, 152.0–704.5) [18]. In a cohort of 659 elective patients without presurgical DVT assessed by ultrasound examination of the lower limbs who underwent gynecologic laparoscopic surgery, 52 developed lower extremity DVT after surgery [19]. Patients who developed DVT had higher serum HE4 levels (median, 45.7 pmol/L; IQR, 34.8–66.1) than the remaining cohort (median, 33.2 pmol/L; IQR, 24.9–45.6), a difference that remained significant in a multivariable-adjusted logistic regression model (*P* = .02) [19].

In an unsupervised proteome-wide discovery approach, we recently reported that plasma HE4 levels were associated with a risk of future VTE in an analysis adjusted for age and sex [20]. However, the relationship between HE4 and VTE may be influenced by putative confounding factors. In the present analyses, we confirmed that elevated HE4 levels were associated with advancing age [21], history of CVD [28], chronic inflammation (assessed by CRP) [59,60], and impaired kidney function (assessed by eGFR) [30], all of which are individually associated with VTE risk [15,38–43] and may therefore act as potential confounders of the relationship between HE4 levels and VTE risk. However, the risk estimates for VTE by HE4 remained essentially unchanged in the multivariable-adjusted models that included all putative confounders. Furthermore, analyses with the start of follow-up 1 or 2 years after blood sampling yielded results similar to those of the overall analyses, indicating that our findings were not explained by underlying occult cancer at the time of blood sampling. Nonetheless, despite the apparent strength of the relationship between plasma HE4 levels and the future risk of VTE, residual confounding cannot be ruled out given the observational design of the study [71].

Mendelian randomization (MR) is a suitable approach to assess causal relationships between exposures and diseases in observational studies, as gene variants (instrumental variables) randomly assigned during gamete production are typically not susceptible to bias from confounding or reverse causation [72–74]. Two previous large proteogenomic studies [75,76] have identified some *trans-* and *cis-*pQTLs that explained a minor proportion (≤1.7%) of the plasma variability in HE4 levels, of which 1 *cis* variant (rs2272953) was detected in both studies and explained only 0.3% of the plasma variability in HE4 [75,76]. In our study, rs2272953 did not reach genome-wide significance (*P* = .53). We identified 2 other *trans-*pQTLs (rs2392864 and rs616530) in linkage disequilibrium, which explained 1.6% of the variability in plasma HE4. However, these SNPs were located in the intron region of LncRNA ENSG00000234464 on chromosome 1, and when conditioning on the lead SNP (rs616530), no genome-wide association was found, further supporting the lack of evidence for genetic regulation of this plasma protein. Due to the inconsistency of potential instrumental variables and their limited ability to explain plasma variability in HE4 across studies, we did not have sufficient instrumental variables to conduct MR and therefore could not further investigate the likelihood of causality for the observed association between HE4 and VTE using MR.

HE4 levels appeared to be most strongly associated with the risk of unprovoked VTE. The stronger association with unprovoked vs provoked events strengthens the inference of a causal association [77], as it indicates that plasma HE4 levels are associated with the risk of future VTE in the absence of known provoking factors. Moreover, elevated HE4 levels exhibited a higher risk of DVT than PE. Although PE and DVT are regarded as 2 entities of the same disease, there are several examples of risk factors with a differential impact on the presenting location of VTE [78]. The most noteworthy is the factor V Leiden (FVL) paradox, in which the FVL mutation increases the risk of DVT but not of PE [79]. Data from a mouse experiment suggest that FVL is associated with enhanced thrombin generation, resulting in larger, more stable thrombi that are less prone to embolization [80]. HE4 may contribute to VTE through similar pathophysiological mechanism(s).

As HE4 appears to be a strong risk factor for VTE independent of major confounders, it is appropriate to consider whether plasma HE4 is either a marker of a prothrombotic environment (innocent bystander) associated with VTE or is directly implicated in its pathogenesis. Despite studies showing that HE4 levels correlate with some hemostatic markers (eg, D-dimer, fibrinogen degradation products, and activated partial thromboplastin time in certain diseases [eg, ovarian cancer and elective laparoscopy in women] [19,81]), we are not aware of any evidence for a direct effect of HE4 on hemostatic factors associated with VTE risk. Moreover, *in vitro* experiments have shown that tumor necrosis factor-α upregulates HE4 expression [82], overexpression of HE4 facilitates the expression of certain inflammatory cytokines [83,84], and various inflammatory diseases are associated with elevated HE4 levels [22,26,27]. Despite evidence supporting associations between inflammation and HE4, and between inflammation and VTE risk [38], chronic inflammation did not seem to explain the relationship between HE4 and VTE, since adjustment for CRP had a marginal effect on the risk estimates.

The strong association between plasma HE4 and GDF-15 levels, as well as their individual association with VTE risk, might reflect a shared response to an underlying regulatory mechanism with prothrombotic properties. GDF-15 is considered a marker of oxidative stress [31,32]. Likewise, growing evidence supports that HE4 is a marker of oxidative stress. First, previous studies [21,22] have reported a strong relationship between smoking and HE4 levels, and in our study, plasma levels of both HE4 and GDF-15 varied according to smoking status. Second, HE4 expression was upregulated in lung tissues of mice exposed to cigarette smoke [22]. Third, cigarette smoke and hydrogen peroxide upregulated HE4 expression in human bronchial epithelial cells, with responses considerably alleviated by N-acetylcysteine, a reactive oxygen species scavenger [22]. Fourth, HE4 levels were inversely related to glutathione levels, an important antioxidant, in the erythrocytes of women with endometriosis [85].

Strengths of the current study include recruitment of participants from a population-based cohort, high-quality biobanking of plasma samples, validation of VTE events, and a clear temporal sequence between plasma protein measurements and the outcome (VTE). Additionally, the SomaScan 7k aptamer-based platform was independently confirmed to be a highly sensitive proteomics assay [86] and to have low variability in HE4 (CV = 5.3%) and GDF-15 (CV = 8.3%) measurements [50]. Furthermore, a correlation of 0.88 was reported for HE4 measurements by SomaScan and isobaric tandem mass tag mass spectrometry in cerebrospinal fluid from patients with Alzheimer’s disease and controls [87]. The study also has potential limitations. Plasma HE4 and GDF-15 levels may have been affected by prolonged storage at −80 °C from study inclusion (2006–2008) to analysis (2022) and are likely to fluctuate in individuals over time [21,88]. Both of these circumstances would presumably lead to regression dilution, resulting in an underestimation of the true association [89]. Although we adjusted for many important confounders, residual confounding could not be completely ruled out due to the observational nature of the study. However, the high E-value (>2) indicates that residual confounding is unlikely to explain our findings. The power of the pQTL analysis was limited due to the small sample size, and the lack of genetic regulation cannot be ruled out. Nonetheless, since MR was not feasible in our study, causality remains to be assessed. The study participants were derived from a cohort of mainly European ancestry (98.8%), and the findings may therefore not be generalizable to other ethnicities.

In conclusion, we found that HE4 and GDF-15 were strongly correlated, and that both proteins were higher in current smokers than in nonsmokers. HE4 levels were associated with the risk of future VTE in a dose-response relationship, and the strength of the association was maintained after adjustment for multiple potential confounders. The strong correlation between HE4 and GDF-15 levels, as well as their association with VTE risk, might reflect a shared response to an underlying regulatory mechanism with prothrombotic properties (eg, oxidative stress). However, further studies are warranted to confirm our findings on the association between plasma HE4 levels and VTE risk and to elucidate the underlying mechanisms.

## Supplementary Material

1

The online version contains supplementary material available at https://doi.org/10.1016/j.jtha.2026.03.009.

## Figures and Tables

**TABLE 1:** Baseline characteristics of the subcohort across quartiles of plasma human epididymis protein 4 levels.

Baseline characteristics	Plasma HE4			
	Q1	Q2	Q3	Q4
*n*	267	266	266	267
Age (y)	54 ± 13	63 ± 10	68 ± 10	73 ± 10
Sex (women)	45.3 (121)	46.2 (123)	45.5 (121)	41.2 (110)
BMI (kg/m^2^)	27.9 ± 4.2	28.1 ± 4.1	27.4 ± 3.9	27.0 ± 4.1
Smoking	7.1 (19)	14.3 (38)	17.7 (47)	28.8 (77)
CRP (mg/L)	1.3 (0.6–2.7)	1.4 (0.7–2.7)	1.5 (0.7–3.0)	2.0 (0.9–7.3)
eGFR (mL/min/1.73 m^2^)	100.7 ± 12.6	92.2 ± 12.7	86.1 ± 13.6	75.0 ± 19.4
History of CVD	0.7 (2)	4.1 (11)	7.9 (21)	9.4 (25)

Data are presented as % (*n*), mean ± SD, or median (IQR).

BMI, body mass index; CRP, C-reactive protein; CVD, cardiovascular disease; eGFR, estimated glomerular filtration rate; HE4, human epididymis protein 4; Q, quartile.

**TABLE 2:** Hazard ratios with 95% CIs for overall venous thromboembolism (VTE), unprovoked VTE, and provoked VTE across quartiles of plasma human epididymis protein 4 levels.

Quartiles of HE4	Subcohort	Cases	HR (95% CI)		
			Model 1	Model 2	Model 3
**Overall VTE**					
Q1	266	50	1 (Reference)	1 (Reference)	1 (Reference)
Q2	266	66	1.47 (0.96–2.25)	1.49 (0.97–2.29)	1.48 (0.96–2.30)
Q3	263	65	1.47 (0.93–2.33)	1.59 (1.00–2.54)	1.59 (0.98–2.58)
Q4	263	113	2.54 (1.60–4.03)	2.87 (1.80–4.59)	2.75 (1.63–4.63)
**Unprovoked VTE**					
Q1	266	17	1 (Reference)	1 (Reference)	1 (Reference)
Q2	266	31	2.07 (1.07–4.00)	2.15 (1.10–4.22)	2.10 (1.06–4.16)
Q3	264	21	1.44 (0.67–3.08)	1.69 (0.78–3.66)	1.62 (0.73–3.60)
Q4	264	46	3.11 (1.50–6.45)	3.81 (1.82–8.01)	3.29 (1.44–7.52)
**Provoked VTE**					
Q1	267	33	1 (Reference)	1 (Reference)	1 (Reference)
Q2	266	35	1.17 (0.69–1.97)	1.16 (0.68–1.96)	1.17 (0.69–2.00)
Q3	265	44	1.47 (0.86–2.54)	1.52 (0.88–2.61)	1.55 (0.88–2.72)
Q4	266	67	2.25 (1.30–3.90)	2.38 (1.37–4.14)	2.43 (1.31–4.51)

Model 1: adjusted for age, sex, and sample batch. Model 2: adjusted for model 1 plus body mass index. Model 3: adjusted for model 2 plus estimated glomerular filtration rate, cardiovascular disease history, C-reactive protein, and smoking.

HE4, human epididymis protein 4; HR, hazard ratio; Q, quartile.

**TABLE 3:** Hazard ratios with 95% CIs for deep vein thrombosis and pulmonary embolism across quartiles of plasma human epididymis protein 4 levels.

Quartiles of HE4	Subcohort	Cases	HR (95% CI)		
			Model 1	Model 2	Model 3
**DVT**					
Q1	267	20	1 (Reference)	1 (Reference)	1 (Reference)
Q2	266	26	1.68 (0.89–3.19)	1.63 (0.86–3.09)	1.47 (0.77–2.83)
Q3	265	23	1.70 (0.84–3.42)	1.80 (0.89–3.62)	1.52 (0.74–3.15)
Q4	264	44	3.51 (1.77–6.97)	3.89 (1.96–7.73)	3.04 (1.42–6.48)
**PE**					
Q1	266	30	1 (Reference)	1 (Reference)	1 (Reference)
Q2	266	40	1.35 (0.79–2.29)	1.40 (0.82–2.39)	1.48 (0.86–2.55)
Q3	264	42	1.33 (0.76–2.34)	1.46 (0.82–2.59)	1.60 (0.88–2.92)
Q4	266	69	2.06 (1.17–3.62)	2.34 (1.32–4.16)	2.54 (1.32–4.85)

Model 1: adjusted for age, sex, and sample batch. Model 2: adjusted for model 1 plus body mass index. Model 3: adjusted for model 2 plus estimated glomerular filtration rate, cardiovascular disease history, C-reactive protein, and smoking.

DVT, deep vein thrombosis; HE4, human epididymis protein 4; HR, hazard ratio; PE, pulmonary embolism; Q, quartile.

## Data Availability

Access to the Trøndelag Health Study (HUNT) data can be obtained by application to the HUNT administration at https://www.ntnu.edu/hunt/research.
