# Supplementary material for: Plasma level of human epididymis protein 4 is associated with risk of future venous thromboembolism—the Trøndelag Health study
Source: J Thromb Haemost. Author manuscript; Available in PMC 2026 Aug 19. (PMC13486973; doi:10.1016/j.jtha.2026.03.009)
Supplement: Supplementary file 1 [file NIHMS2192018-supplement-1.docx]

**Supplementary Tables**

**Supplementary Table 1**. **Baseline characteristics of the case-cohort.**

|  | VTE cases n=294 | Subcohort n=1,066 |
| --- | --- | --- |
| Age (years), mean ± SD | 66 ± 15 | 64 ± 13 |
| Sex (women), % (n) | 51 (151) | 45 (475) |
| BMI (kg/m^2^), mean ± SD | 28.4 ± 4.7 | 27.6 ± 4.1 |
| Smoking, % (n) | 18 (52) | 17 (181) |
| CRP (mg/L), median (IQR) | 1.6 (0.8-3.7) | 1.5 (0.7-3.1) |
| eGFR (ml/min/1.73m^2^), mean ± SD | 84.2 ± 20.4 | 88.5 ± 17.5 |
| History of CVD, % (n) | 7 (20) | 6 (59) |

BMI, body mass index; CRP, c-reactive protein; CVD, cardiovascular disease; eGFR, estimated glomerular filtration rate; IQR, interquartile range; SD, standard deviation; VTE, venous thromboembolism.

**Supplementary Table 2. Characteristics of patients with incident VTE (n=294) at diagnosis.**

| VTE characteristics | VTE cases |
| --- | --- |
| Age at VTE, mean ± SD | 68 ± 15 |
| DVT, % (n) | 38 (113) |
| PE, % (n) | 62 (181) |
| Unprovoked VTE, % (n) | 39 (115) |
| Provoked VTE, % (n) | 61 (179) |
| Cancer associated VTE, % (n) | 19 (56) |

DVT, deep vein thrombosis; PE, pulmonary embolism; SD, standard deviation; VTE, venous thromboembolism.

**Supplementary Table 3. Characteristics of the two genome-wide significant SNPs identified in the protein quantitative trait loci (pQTL) analysis.**

| **ID** | **CHR** | **POS** | **REF** | **ALT** | **MAF** | **AF** | **CHISQ** | **BETA** | **SEBETA** | **PVALUE** | **LOG10P** | **INFO** | **N** | **RSID** |
| --- | --- | --- | --- | --- | --- | --- | --- | --- | --- | --- | --- | --- | --- | --- |
| 1:238726592_C/T | 1 | 238726592 | C | T | 0.211268 | 0.211268 | 29.8218 | -0.218664 | 0.0400415 | 4.7e-08 | 7.32790214206428 | 0.99705 | 1900 | rs2392864 |
| 1:238727663_T/A | 1 | 238727663 | T | A | 0.215544 | 0.784456 | 29.8989 | 0.21753 | 0.0397824 | 4.6e-08 | 7.33724216831843 | 0.9986 | 1900 | rs616530 |

**Supplementary figure 1: Spline plots displaying the hazard ratio (HR) of overall venous thromboembolism (VTE) as a function of plasma human epididymis protein 4 (HE4) levels in a generalized additive regression model. Panel A shows sensitivity analyses with follow-up started one year after blood sampling, while Panel B shows sensitivity analyses with follow-up started two years after blood sampling. The HRs were adjusted for age, sex, sample batch, BMI, eGFR, CVD history, and CRP. The black lines depict the HRs while the white lines represent the quartile cut-offs. The dark grey areas represent the 95% CIs while the light grey areas are density plots showing the distribution of plasma HE4 levels. BMI, body mass index; CI, confidence interval; CVD, cardiovascular disease; CRP, C-reactive protein; eGFR, estimated glomerular filtration rate.**

**Supplementary figure 2: Conditional protein quantitative trait loci (pQTL) with rs616530 as covariate. A Manhattan plot of pQTL analysis (GRCh37/hg/19 was used as reference human genome). The upper, dashed red line indicates the genome-wide significance level threshold of p<5×10-8 for associations with the plasma variability of human epididymis protein 4 (HE4) levels.**
